# Pulmonary toxicity in patients treated with gemcitabine and a combination of gemcitabine and a taxane: investigation of a signal using postmarketing data

**DOI:** 10.1038/sj.bjc.6603161

**Published:** 2006-05-09

**Authors:** A Czarnecki, S Voss

**Affiliations:** 1 Eli Lilly and Company Limited, Erl Wood Manor, Sunninghill Road, Windlesham, Surrey GU20 6PH, UK

**Sir**,

We were interested to read a recent article by Vasey *et al* (2006) in which the authors expressed some concern about the incidence of pulmonary toxicity (Ptox) in patients treated with a taxane–gemcitabine regimen, an issue that has been raised by several others (Thomas *et al*, 2000; Bhatia *et al*, 2002; Harries *et al*, 2004). The rare specific adverse drug reactions (ADRs) classified as Ptox are known to occur with the use of gemcitabine (Gem) and both taxanes, docetaxel (Doc) and paclitaxel (Pac). All three drugs are used as single agents and increasingly in combination treatment.

In order to evaluate whether these combinations have greater Ptox than single agents, the Eli Lilly spontaneous safety database (LSD) was searched for MedDRA terms (pulmonary oedema, acute respiratory distress syndrome (ARDS), dyspnoea and pneumonitis NOS) for Gem and Gem+taxane combinations. All of these reactions are listed in the relevant product information. Identified Ptox reports were reviewed individually and as aggregate data. In addition, Proportional Reporting Ratios (PRRs) were calculated from the FDA Freedom of Information (FDA-FOI) database; similar PRR calculations were also performed from the LSD (12 January 1998 to 30 June 2004). Proportional Reporting Ratios compare rates of events observed with one particular drug with the rates of the same events that may be expected with exposure to all drugs in the database (Evans *et al*, 2001) and reflect the strength of association between a drug and an event.

Pulmonary toxicity ADRs reported to Lilly in patients treated with Gem+taxane combinations were compared to those reported in patients receiving Gem monotherapy. An aggregate review did not provide evidence of a different Ptox safety profile for Gem used alone or in combination with taxanes. PRRs from the LSD for Gem+taxane combinations and Gem alone showed a similar trend (data not shown), which could indicate similar Ptox profiles for all three drugs.

The PRR calculations for Ptox for Gem+taxane combinations and for Gem and the taxanes alone, calculated from the FOI database, show broadly similar patterns for the terms that are included in the labels of all three drugs (ARDS Gem 9.37, Doc 6.81, Pac 1.97; Pulmonary Oedema Gem 2.20, Doc 1.99, Pac 2.64; Dyspnoea Gem 3.09, Doc 1.79, Pac 1.61; Pneumonitis NOS Gem 9.52, Doc 9.63, Pac 3.70). It must be noted that PRRs reflect the strength of association but there is no linear relationship between the PRR value and the association between drug and event; values over 1 indicate the association and values of at least 2 are generally accepted as indicating a possible safety signal (Evans *et al*, 2001). Any potential for additive toxicity of the drugs used in combination (specifically for pneumonitis) may be due to both components (Gem+Doc ARDS 4.32, Pulmonary Oedema 3.07, Dyspnoea 1.91, Pneumonitis 21.94; Gem+Pac ARDS 1.03, Pulmonary Oedema 2.32, Dyspnoea 2.08, Pneumonitis 15.97). The FOI database contains larger numbers of drugs and events, and therefore higher numerators and denominators, than the LSD that results in lower ratios in general.

The review of Ptox cases indicated that there was no substantial difference in Ptox reported with the combination of Gem and taxanes in comparison to Gem alone. The strength of association (PRRs) with respect to Ptox calculated on data from the LSD and the FDA-FOI database support this finding. In addition, PRR calculations for taxanes on the FDA-FOI database seemed to indicate a similar safety profile (PTox) for all three drugs. Therefore, any potential increase in toxicity of these drugs used in combination seems to be due to both components of the combination (additive toxicity). The PRRs were calculated here for taxanes with and without gemcitabine, they were not calculated for combinations of taxanes and other chemotherapeutic agents.
